# ICD coding of death certificates with generative language models

**DOI:** 10.1371/journal.pdig.0001245

**Published:** 2026-02-24

**Authors:** Isabel Coutinho, Gonçalo M. Correia, Bruno Martins, Afonso Moreira, André Peralta-Santos

**Affiliations:** 1 INESC-ID, Lisbon, Portugal; 2 Instituto Superior Técnico, University of Lisbon, Lisbon, Portugal; 3 Priberam, Lisbon, Portugal; 4 Direção-Geral da Saúde, Lisbon, Portugal; Harvard Medical School, UNITED STATES OF AMERICA

## Abstract

Although large language models can achieve remarkable results in most text generation tasks, these models have been less used in text classification problems, of which ICD coding of clinical documents is one example. In this work, we propose different strategies to adapt a LLaMA generative language model to the ICD coding task. In one such strategy, we only use a language modeling objective for training, followed by constrained decoding at inference time, rather than fine-tuning the model for discriminative classification. We specifically use free-text descriptions in Portuguese death certificates to train a relatively small LLaMA model for assigning ICD codes to the underlying cause of death, and we compare it against a BERT encoder model, which is typically used to address text classification tasks. Experiments show that generative language models can achieve strong results in ICD coding of death certificates, with a classification accuracy that is at least in line with the results obtained using encoder models. We thus demonstrate that language generation can be a suitable approach for ICD coding, allowing for multiple related tasks, such as coding the underlying or the multiple causes contributing for a death, to be performed with a single unified model.

## Author summary

The ICD coding system corresponds to a standardized way of classifying health conditions and external causes of injury or disease. Assigning these codes to causes of death is a critical task in public health, in order to monitor a population’s health and to conduct mortality and morbidity studies. However, manually coding death certificates is time-consuming and error-prone. Our work explores whether modern language models can help automate the ICD coding task effectively. We specifically frame ICD coding as a language generation problem, where human coders can interact with the model with a simple language prompt, allowing for different tasks to be performed with a single unified model, such as coding the underlying cause or multiple causes of death. We achieved strong experimental results, showing that this solution has indeed the potential to be applied in a real-life public health surveillance scenario, either in a setting where it is only necessary to obtain approximate mortality statistics, or in a setting where human coders can refine the automatically generated results, but considerably alleviating the burden to public health professionals.

## 1 Introduction

The International Classification of Diseases (ICD) coding system, proposed by the World Health Organization, corresponds to a standardized way of classifying health conditions and external causes of injury or disease. Although this standard has been adopted worldwide in the medical domain, manually assigning ICD codes to clinical text is still a time-consuming and error-prone task [1]. In the particular context of coding death certificates, it is essential that this task is performed in a precise and efficient way, to monitor a population’s health and to conduct mortality and morbidity studies. Thus, as a way of addressing the challenges associated to manual coding, many efforts have already been placed in the devolvement of automated coding approaches [2].

Despite the recent interest in Large Language Models (LLMs) for clinical natural language processing, and despite their exceptional performance in text generation tasks, these models are usually not suited for the ICD coding task without the introduction of several modifications. In fact, previous work has argued that LLMs can hardly outperform encoder-only models, e.g. based on the BERT [3] or RoBERTa [4] neural network architectures, in different classification tasks [5].

In this work, we present different strategies to address the ICD coding task with generative language models, highlighting a model that requires a single training phase with a language modeling objective, followed by constrained decoding at inference time to ensure the generation of a valid ICD-10 code description.

We specifically analyzed free-text descriptions in death certificates, along with the associated autopsy reports and clinical bulletins, in order to determine the ICD code for the Underlying Cause of Death (UCOD), formulating this task as either a discriminative multi-class classification problem, or as a text generation problem. Similarly to previous work [6,7], we used data from the Portuguese Directorate-General of Health (DGS), but in this case we considered a much larger dataset that includes certificates from the years 2014 to 2021, thus assessing results in a more challenging classification scenario. We conducted several experiments with a LLaMA generative language model [8], comparing it against a BERT encoder model. We obtained strong experimental results, showing that language generation can be a suitable approach for ICD coding, with a classification accuracy that is at least in line with the results obtained using encoder models, but allowing for different tasks to be performed with a single unified model (e.g., multi-label classification of multiple causes of death). This means that, instead of relying on separate models or modules for each specific task, a single model can handle multiple tasks as language generation, by adapting its behavior according to the user’s prompt.

We also demonstrate that when formulating the ICD coding task as a text generation problem, with appropriate strategies, we can obtain a probability distribution over the various possible ICD codes, as would be the case with traditional encoder models. This allows us to explore different uses of the model and different applications related to real-life public health surveillance. For instance, we can use conformal prediction [9], which is a simple approach for creating statistically rigorous uncertainty sets for the predictions of a model, to assign individual instances to sets of ICD codes that are guaranteed to contain the ground-truth code with a given coverage. We can also use the probabilities to better estimate the frequency of ICD codes in a given unlabeled dataset (i.e., the problem of text quantification [10,11]), summing the posterior probabilities assigned to each class by the model rather than simply counting the output classes for each individual input in the dataset.

The rest of this document is organized as follows. Sect 2 outlines previous related work on automatic ICD coding. Sect 3 describes the proposed strategy for training language models for ICD coding. Sect 4 presents the main experiments and the results that were obtained, ending with a discussion on how can we explore text generation for addressing other tasks besides simple multi-class ICD coding. Finally, Sect 5 summarizes the main conclusions and presents possibilities for future work in the area.

## 2 Related work

Several previous studies have already addressed the problem of automatic ICD coding, generally formulating the task as a discriminative multi-label classification problem [2]. The majority of previous experiments have been conducted using a publicly available dataset, namely MIMIC-III [12], which comprises information relating to patients admitted to critical care units, specifically analyzing hospital discharge summaries and aiming to assign a set of ICD diagnosis and procedure codes per instance.

Pre-trained Language Models (PLMs) using the Transformer encoder architecture [13] usually achieve state-of-the-art results on the ICD coding task. An example of one such previous approach is PLM-ICD [14]. The authors identified the main challenges faced when applying PLMs to ICD coding, and they proposed different mechanisms to tackle them, namely a domain-specific pre-training stage for addressing the domain mismatch problem (i.e., clinical notes can use medical-specific terminology, thus being very different from the general-domain text corpora commonly used for pre-training), segment pooling for handling long text as input, and also a label attention mechanism to deal with the large set of possible ICD codes that can be assigned.

Another relevant work in the area was reported by Gomes et al. [15]. In this case, the authors started from a pre-trained model on the healthcare domain, and they advanced two different approaches to process long clinical narratives, either using an encoder with a sparse attention mechanism, or dividing the text into chunks and processing each one independently. These authors also proposed a multi-synonyms attention mechanism, which consists of a label attention mechanism that leverages synonyms to improve the representation of each ICD code, thus aiding in ICD classification.

Studies specifically addressing the classification of death certificates, particularly considering non-English data, are more scarce. One example is the work from Falissard et al. [16], proposing a deep neural network model to predict the ICD-10 code for the UCOD from French death certificates, leveraging detailed textual descriptions and also structured data (i.e., age, gender, and year of death). Although the authors proposed a simple method based on a convolutional neural network, the analysis of the results is particularly interesting. The authors compared their method with the IRIS software (i.e., a well-known rule-based system for automatic coding of multiple causes of death and for the selection of the UCOD), considerably outperforming it. The authors also presented a detailed error analysis and described a practical application regarding the analysis of overdose-related deaths, highlighting the model’s potential for automating mortality data classification and facilitating public health surveillance.

A more recent study from Zambetta et al. [17] proposed a different solution for the ICD classification problem of French death certificates, by combining three coding methods, namely a deep learning approach, a rule-based system, and also manual coding. First, a Transformer encoder-decoder model, designed for tasks that involve mapping input sequences to output sequences, predicts the ICD code for each of the fields of the certificate (i.e., multiple causes of death) and for the UCOD. A confidence value is also computed for each certificate, allowing certificates with less confidence to be manually coded. In the second step, the training data is updated with the new instances given by the manual coding, and the model is retrained on these data. Additionally, it is possible to use one of the IRIS software modes, in which given the ICD codes corresponding to the conditions reported on the death certificate, the system selects the UCOD. Consequently, there are potentially multiple suggestions for the main cause of death (i.e., two coming directly from the Transformer model, and two other coming from IRIS). In the final step, a Bi-LSTM neural module performs a classification task and chooses between the different code proposals.

To the best of our knowledge, the work from Duarte et al. [6] which used recurrent neural networks, the subsequent validation study from Ferreira et al. [18], and the more recent work from Coutinho and Martins [7] which used a BERT encoder model, are the only studies focusing on coding death certificates using the Portuguese language. Although these studies also considered data provided by the Portuguese DGS, our work uses a much larger dataset, thus assessing results in a more challenging classification scenario. We also use additional information regarding the gender and the age of the deceased individuals, as well as some annotations from the health professionals that filled the reports, which can include important details to aid in code selection. Additionally, whereas in previous work the different fields of the death certificates were directly feed into to the neural network models, we now first transform each instance into a properly formatted textual prompt, with natural language descriptions for each of the fields.

## 3 Methods

In this work we explored the use of LLaMA-3.2 multilingual language models, developed by Meta AI [8], adjusting their parameters through supervised training so as to properly represent Portuguese death certificates. LLaMA refers to a family of generative language models based on the Transformer decoder architecture [13], trained using publicly available datasets and made available in open-access.

LLaMA models are designed to support various generalist natural language generation tasks, e.g. question-answering, summarization, or translation, achieving state-of-the-art results in several benchmarks. A less explored option is to adapt these models to discriminative tasks such as text classification. In fact, for different types of text classification tasks, even with careful prompt engineering and/or instruction-tuning techniques, these models can hardly outperform encoder-type models such as BERT [3] or RoBERTa [4], particularly without additional adaptions in the model’s architecture [5].

In our experiments, we considered two distinct phases for model training, namely *continual pre-training* and *fine-tuning*. We start by pre-training the model in order to adapt it to the clinical domain, using causal language modeling. The continuously pre-trained model can be used to address the ICD coding task as a text generation problem, or alternatively we can further fine-tune the model into a discriminative classifier. In this second case, the pre-trained model parameters are further fine-tuned using labeled data for the classification task, together with a small task-specific layer. We detail each phase in the next sections.

### 3.1 Continual model pre-training

For continual pre-training, we started by adapting the dataset of Portuguese death certificates, which includes multiple text fields together with structured data such as age and gender, and also clinical bulletins and autopsy reports, into a textual format (see Sect 4.1 for more details about the dataset). We designed a collection of rules, in order to transform each instance of the dataset into a set of properly formatted textual prompts in Portuguese. Each prompt contains a short contextual description about the ICD coding scenario, and all available information about the deceased individual, along with a task instruction and an expected output. We further describe the structure of the textual prompts in S1 Appendix.

The model was then trained considering a causal language modeling objective, i.e., predicting the next token in a sequence given the previous tokens. The common approach, which we also followed, is to minimize the standard cross-entropy loss. Following recent work, we applied the loss function to all the tokens of each instance, rather than solely to the tokens of the expected output [19].

We used this model for addressing the ICD coding task as a text generation problem, by using constrained decoding to control the output of text generation and ensure it is a valid ICD code. We thus force the model to fulfill this constraint, by verifying allowed tokens at every step of text generation.

### 3.2 Model fine-tuning

The fine-tuning of the LLaMA-3.2 model as a discriminative classifier involves minimal changes in the architecture, adding a classification head over a pooled representation from the prompt, which consists of a fully-connected layer that processes the embedding of the input and projects it into the label space for classification. Since the ICD coding task is formulated as a multi-class classification problem, in which the objective is to predict the single ICD code for the UCOD, we also use the softmax activation function over the logits given by the fully-connected layer (i.e., in order to normalize the scores and obtain the probability of each ICD code to be assigned), and the categorical cross-entropy loss function. All parameters of the language model, along with the classification head, are learned by minimizing the loss function over the training set.

It is important to notice that generative language models have unidirectional context, in the sense that they only consider the past and not the future context when generating predictions from sequences of text. We address this limitation following the work of Springer et al. [20], specifically repeating the input text twice, so that the “echo” embeddings, namely the representations of the tokens in the second part of the prompt (i.e., the repetition), can encode information about the complete prompt. We can thus emulate a bidirectional self-attention mechanism when building input representations.

We compute the embedding of the input from the activations of the final hidden layer of the decoder. Considering only the tokens of the second part of the prompt, each input token *x*_*i*_ at position *i* is associated with a contextualized token embedding given by the hidden layer representation $\phi (x_{i})$. To create a pooled representation of the input, instead of considering one common strategy that simply consists of computing the mean token embedding over the set of tokens, we use a weighted average. Given the second part of the prompt with *T* tokens, denoted as $X_{2nd}=\{x_{1},x_{2},...,x_{T}\}$, the weighted average of the token embeddings is represented as follows:

$$\phi_{document}=\sum_{i=1}^{T}w_{i}\phi (x_{i}),$$ (1)

in which $w_{i}=\frac{i}{\sum_{i=1}^{T}i}$ corresponds to the weight of each token. The tokens at the end of the prompt are given more importance than the initial ones.

## 4 Experimental evaluation

This section describes the experimental evaluation of the aforementioned alternative approaches for ICD coding. We present a statistical characterization of the dataset used in our tests (Sect 4.1), the details regarding the experimental setup (Sect 4.2), and the results that were obtained (Sect 4.3).

### 4.1 Dataset

The dataset used in our experiments corresponds to the death certificates submitted to the Portuguese Death Certificate Information System (SICO) from the years 2014 to 2021. Due to confidentiality constraints, the dataset cannot be made publicly available, although interested researchers can contact the Portuguese DGS.

**Fig 1:**
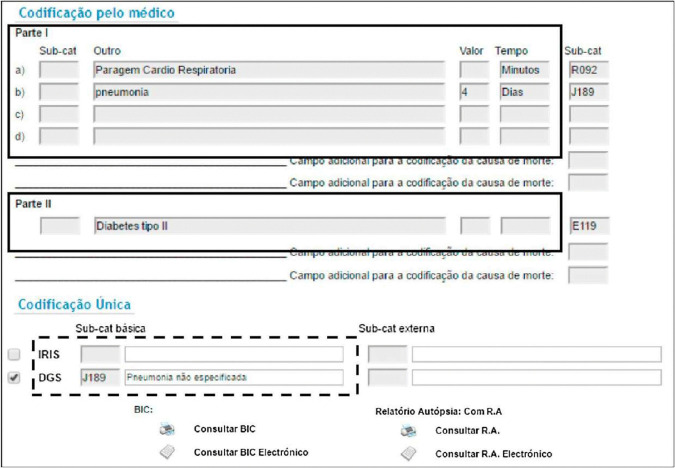
The electronic form used in Portugal for death certificate registration and subsequent manual ICD coding [6], highlighting Parts I and II of the form, plus the area for coding the UCOD.

The online form presented by SICO consists of two parts: Part I (up to 4 fields) describes the chain of events leading directly to death, whereas Part II (optional) reports other conditions that contributed to death, but that are not part of the causal sequence of events that lead to the death. Clinical bulletins and autopsy reports can be associated with a death certificate in cases of deaths caused by violent or unknown causes. Some additional annotations written by the medical coders can be added, which in many cases may include important details, and we also have information regarding the gender and the age of the deceased individual. Together with this information, there is an ICD-10 code corresponding to the UCOD assigned a posteriori by specialized clinical coders, and there may also be a set of ICD-10 codes for other auxiliary causes. Fig 1 presents a screen-shot of the online form presented by SICO to collect a death certificate.

We divided the data into two parts for experiments: the main set and an additional set comprising data of the last six months of 2021. The experiments with the main dataset considered a total of 843,103 instances. We divided the main set into training, validation, and testing splits in a stratified manner, i.e., according to the frequency of the ICD-10 codes, ensuring, whenever possible, that the distribution of each code across the three splits was 80%, 10%, and 10%. We guaranteed that each ICD code was seen at least once during training.

The additional set, used to better assess model generalization capabilities and to evaluate performance in a possible real-life public health surveillance scenario, considered a total of 57,089 instances. We exclude the ICD-10 codes (and associated instances) that were not present in the main set (i.e., a total of 64 distinct codes). Table 1 includes the main dataset statistics, comparing them with the dataset used in previous work [6,7].

**Table 1:** Descriptive statistics for the dataset used in our experiments, together with a comparison with a similar dataset used in previous work [6,7].

Description	Our dataset	Previous dataset
Number of instances in the main set	843,103	121,536
Number of instances in the training set	674,508	91,152
Number of instances in the validation set	84,303	—
Number of instances in the test set	84,292	30,384
Number of instances in the additional set	57,089	86,071
Number of distinct ICD-10 full-codes in the main set	3,644	1,418
Number of distinct ICD-10 blocks in the main set	1,131	611
Number of distinct ICD-10 chapters in the main set	21	18

**Fig 2:**
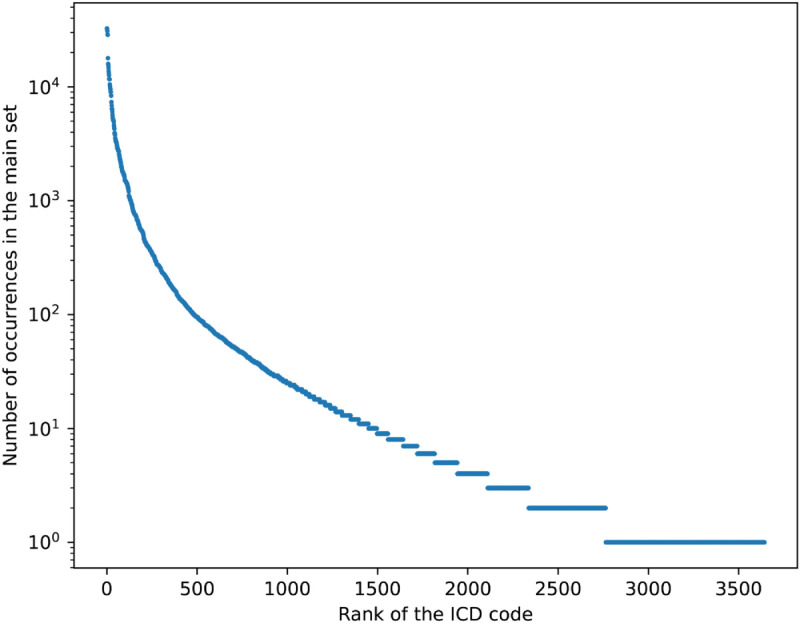
Number of occurrences of the ICD codes in the dataset (in log scale) for each ICD code in the main set.

One of the main challenges regarding these data relates to the label distribution being extremely imbalanced. In fact, this problem was already present in a version of the dataset used in previous work [6,7], covering an earlier time period. However, in this case we have more than 2 times the number of distinct ICD-10 codes considered previously, and thus the long-tail is even more pronounced. Fig 2 shows the distribution for the ICD codes in this new dataset. The codes are sorted by frequency and the values are plotted on a logarithmic scale, allowing both frequent and rare labels to be featured on the same plot, which would otherwise be dominated by the few highly frequent labels.

### 4.2 Experimental setup

Following previous work [7], our experiments used a Portuguese BERT model as a baseline, namely BERTimbau-Large [21]. This model has 335M parameters and it was pre-trained on the Brazilian Wikipedia and the brWaC corpus [22]. We further adjusted the parameters by running an additional pre-training phase, and then fine-tuned the model for the ICD coding task. The pre-training phase consists of masked language modeling, in which 15% of the tokens are randomly masked and the goal is to predict the original tokens, by minimizing the cross-entropy loss. Fine-tuning is made with a model architecture composed of BERT plus a fully-connected layer that processes the [CLS] token that summarizes the input, also considering the cross-entropy loss function. We used the same dataset for model pre-training and fine-tuning, and we transformed each instance of the dataset into a properly formatted textual prompt for classification (as further described in S1 Appendix).

Regarding the LLaMA models, we considered the latest version at the time in which the experiments were made, i.e. LLaMA-3.2, and we included in our experiments results with the models featuring 1B and 3B parameters. We specifically considered the instruction-tuned versions of the models. The choice of these specific model sizes was related to the available computational resources, since the models must be executed on servers that are located on-premise, due to data privacy constraints. We did not conduct further experiments with larger models, although our tests also pointed to comparable results with the 1B and 3B versions, thus limiting the interest in tests with larger models.

In regard to the experiments with constrained decoding, we report the results with a sampling approach for decoding, considering the top *k* = 10 highest probability vocabulary tokens. For ICD classification, we also returned *k* independently computed sequences (i.e., sequences of tokens that represent an ICD code) and then re-ranked the different codes according to the log probability of each sequence (i.e., the logarithm of the probability of the sequence being generated), which was computed by summing the log probabilities of each token and dividing by the total number of tokens in the sequence. In order to obtain a probability distribution across all ICD labels, we used a softmax activation function over the log probabilities of each sequence (and we give zero probability for the codes outside the *k* returned sequences). We also considered temperature scaling in the softmax function, with the value *T* = 0.01.

All our experiments used an effective batch size of 32 for model training. For the experiments with LLaMA models, we used a single NVIDIA A100 with 80GB, while the experiments with the BERT model are compatible with more modest hardware (e.g., 32GB of GPU memory). We continuously pre-trained the models for a maximum of 5 epochs, monitoring the loss function over the validation set. Additionally, we fine-tuned all models for a maximum of 20 epochs, in this case monitoring the macro-F1 over the validation set. In both training phases, we set the early stopping criteria with a patience level of 2. We also used the AdamW optimizer [23] with a constant learning rate of 2e-5 for all the experiments.

For assessing the quality of the model predictions, we compared the automated results with ICD-10 data manually annotated by human coders from the Portuguese DGS, and measured results in terms of accuracy and macro-averaged precision, recall, and F1-score. The macro-averaged values are computed by averaging metrics computed per-label. Given the hierarchical organization of the ICD codes, results were also measured according to the three different levels of specialization: chapters (i.e., groups of diseases), blocks (i.e., the first three-digits of each ICD code), and the full-code.

### 4.3 Results

We comprehensively evaluated the proposed approaches, comparing them against previous work and also exploring different settings to assess model performance in real-life public health surveillance scenarios.

#### 4.3.1 Main experiments.

We started by comparing BERTimbau-Large, considered as a baseline, against two different variants of a LLaMA-3.2, namely one model with 1B and another with 3B parameters. We also assessed the importance of introducing a continual pre-training phase, in which we first adapt models to the clinical domain, and then fine-tuned them for the classification task. Finally, we compared the fine-tuning alternative with an approach that uses the pre-trained model to tackle the ICD coding task as a text generation problem. In this case, we used the LLaMA-3.2-1B pre-trained model together with a constrained decoding procedure that controls the output of text generation. Table 2 presents the results for the different experiments.

**Table 2:** Results obtained with BERT and LLaMA models. The values in bold represent the best result on each metric and for each level of specialization in ICD.

Model	ICD level	Accuracy	Macro-averages		
			Precision	Recall	F1-score
BERTimbau-Large (fine-tuned)	Chapter	92.4	74.1	69.9	71.4
	Block	85.6	35.0	33.2	33.2
	Full-code	80.8	20.1	20.4	19.5
LLaMA-3.2-1B-Instruct (fine-tuned)	Chapter	92.3	75.5	72.5	73.5
	Block	85.5	35.7	32.7	33.1
	Full-code	80.7	20.7	20.2	19.6
LLaMA-3.2-3B-Instruct (fine-tuned)	Chapter	92.3	74.2	71.7	72.7
	Block	85.5	36.1	33.8	33.9
	Full-code	80.6	20.6	20.4	19.7
BERTimbau-Large (pre-trained + fine-tuned)	Chapter	92.3	70.7	69.2	69.8
	Block	85.4	35.0	33.5	33.3
	Full-code	80.6	20.7	21.0	20.1
LLaMA-3.2-1B-Instruct (pre-trained + fine-tuned)	Chapter	92.4	74.0	72.2	72.8
	Block	85.6	36.4	34.5	34.4
	Full-code	**81.1**	**21.6**	**21.7**	**20.7**
LLaMA-3.2-1B-Instruct (pre-trained + constrained decoding)	Chapter	**92.5**	**75.7**	**75.2**	**75.3**
	Block	**85.7**	**36.8**	**35.5**	**35.1**
	Full-code	80.6	21.0	21.0	20.0

Both LLaMA models present a slightly higher performance when compared with the BERT model, in most evaluation metrics. Adding a continual pre-training stage, which first adapts models to the clinical domain, is also beneficial to classification performance.

Regarding the approach in which we simply use constrained decoding, we first note the strong empirical results, slightly outperforming the other approaches at the chapter and block levels of specialization. Although the evaluation metrics are slightly inferior to those of the fine-tuned model for the case of assigning full-codes, the difference is not particularly significant. We considered this model as the “best” alternative, and we further detail its performance next.

Table 3 specifically shows the results obtained when using the “best” model for assigning full-codes within each of the ICD-10 chapters. The results were obtained considering subsets of the original test set, containing only the instances of the respective chapter. Chapter II (i.e., *neoplasms*) represents approximately 24.98% of the total number of code instances occurring in the main set, while Chapter IX (i.e., *diseases of the circulatory system*) represents 29.41%. In fact, these sets of ICD code instances together represent more than 50% of the instances. As expected, our model achieves significantly better results in these highly frequent chapters, compared to the overall performance, due to the large number of occurrences in the training data.

**Table 3:** Number of instances and results for the “best” model from Table 2, for the full-codes within each of the ICD-10 chapters. The column named “Percentage” corresponds to the percentage of instances associated to a code within specific ICD-10 chapter, considering the occurrences in the training, validation, and test sets.

Chapter	Occurrences			Percentage	Accuracy	Macro-averages		
	Training	Validation	Test			Precision	Recall	F1-score
I	12,133	1,520	1,512	1.80	58.7	18.8	16.9	17.1
II	168,491	21,048	21,064	24.98	90.7	39.1	37.7	37.1
III	2,705	346	332	0.40	62.1	28.2	21.9	23.5
IV	32,822	4,104	4,109	4.87	55.7	30.6	26.5	27.1
V	26,947	3,369	3,367	3.99	82.5	16.7	11.2	12.8
VI	23,920	2,987	2,993	3.55	87.2	31.0	27.7	28.1
VII	8	0	0	0.00	–	–	–	–
VIII	50	5	6	0.01	66.7	20.8	25.0	22.2
IX	198,400	24,802	24,789	29.41	78.4	36.2	32.4	32.8
X	74,905	9,359	9,365	11.11	86.5	36.6	32.5	33.7
XI	29,347	3,665	3,668	4.35	79.7	41.0	36.8	37.5
XII	1,667	206	205	0.25	51.7	26.7	20.4	22.5
XIII	2,867	354	360	0.42	55.6	21.7	15.8	17.5
XIV	20,107	2,515	2,518	2.98	76.3	26.4	21.1	22.4
XV	46	7	4	0.01	33.3	1.8	2.6	2.1
XVI	106	12	8	0.01	62.5	6.0	6.3	5.9
XVII	989	122	124	0.15	50.0	7.9	7.8	7.5
XVIII	34,051	4,260	4,254	5.05	92.3	34.0	27.1	28.7
XIX	1	0	0	0.00	–	–	–	–
XX	30,446	3,809	3,801	4.51	44.1	8.2	7.8	7.0
XXI	0	0	0	0.00	–	–	–	–
XXII	14,500	1,813	1,813	2.15	93.5	50.0	47.1	48.5

**Fig 3:**
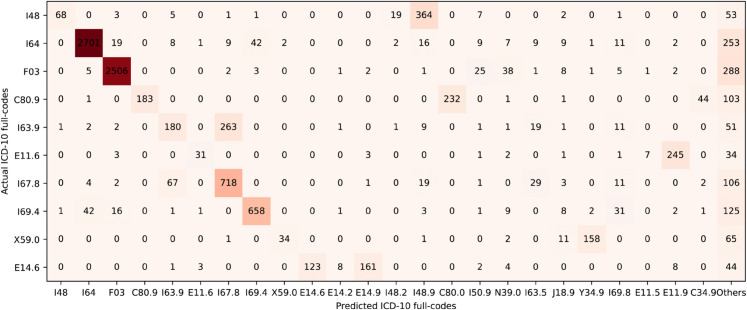
Confusion matrix for the 10 most confused ICD-10 full-codes.

In turn, Fig 3 represents a confusion matrix with the 10 most confused full-codes of the dataset, i.e., the ICD-10 codes with the highest number of errors made by the model (in absolute terms). According to the figure, some of these codes (e.g., I48, E11.6, C80.9, and E14.6) are mainly confused with another code belonging to the same block. In particular, a common confusion is with codes featuring the digit 9 after the block. These are generic catch-all codes that are very similar to the higher-level code (i.e., the corresponding block), including conditions which are not otherwise specified. Differently, the codes I64, I63.9, I67.8, I69.4, and X59.0 are mostly confused with codes from the same chapter, but not necessarily from the same block.

#### 4.3.2 Assessing biases in the classification model.

We also evaluated possible biases in the “best” classification model, grouping individuals by gender and age group, and measuring the classification results within each of the considered groups. Table 4 summarizes the obtained results.

**Table 4:** Number of instances and results for the “best” model from Table 2, for the full-codes within each gender or age group. The number of occurrences by gender or age group does not correspond extacly to the total number of instances in each dataset split, since there are death certificates without this information.

Gender/Age group	Occurrences			Percentage	Accuracy	Macro-averages		
	Training	Validation	Test			Precision	Recall	F1-score
Male	338,492	42,256	42,366	50.19	80.6	18.1	18.3	17.4
Female	336,001	42,045	41,923	49.81	80.6	16.7	17.1	16.2
Under-25	3,127	413	391	0.46	52.9	2.3	2.5	2.3
25-50	23,140	2,923	2,824	3.43	72.8	8.5	8.9	8.3
50-75	16,7441	20,945	20,871	4.82	81.5	14.9	15.4	14.5
75-100	46,9753	58,643	58,813	69.64	80.9	17.5	17.8	16.9
Above-100	6,167	763	801	0.92	84.5	2.7	2.9	2.7

In terms of a the gender-specific analysis, we see no notable difference between male and female individuals in terms of the model’s performance, both obtaining approximately equal accuracy. In what regards the age of the individuals, we divided the test set into five age groups (i.e. under-25, 25-50, 50-75, 75-100, above-100), and saw that the two youngest age groups have lower accuracy than the general population, particularly the under-25 group, with an accuracy of around 53%. Although this value is very low, the number of deceased individuals in this age group is also low (i.e., less than 400 in the test split), and therefore the impact on the overall results is not very significant.

#### 4.3.3 Comparison with previous work.

We also compared the “best” model against previous related work [7], in which a BERT model was used to assign ICD-10 codes for causes of death reported on Portuguese death certificates. As already described, the dataset used in the previous study comprises a much smaller set of ICD codes, thus corresponding to a simpler classification task. Therefore, we evaluated our model in a setting that comprises only the 1,418 codes contained in the dataset from previous work, by running a new inference phase and constraining the decoding to only these ICD codes. In fact, only one code from the previous dataset is not considered in our new label set, as a result of being a rarely used code. However, we can still support the assignment of this code, since our “best” model was not trained as a discriminative classifier, and thus it can also generate the descriptive title of that specific ICD code. Also note that we are not able to establish a direct comparison with previous work, due to differences in the data being used, although we believe that the assessment scenario being reported is fair.

Furthermore, we also evaluated our model in a scenario with the top 2,000 most frequent ICD codes. We identified the 1,999 most frequent codes, and the other remaining codes were all included in a class designated as *Others*. In this case, most of the ICD codes in the previous dataset are included in the top 2,000 (i.e., only 44 codes out of the 1,418 are not included in the top 2,000).

Table 5 presents the results across the different metrics and levels of specialization for the two aforementioned scenarios. We can see that, in the setting where the label set is the same, our model has considerably superior performance. The 1,418 ICD codes used in this test correspond to the ground-truth labels of 96.2% of the instances in the main test set considered in Table 2, thus further demonstrating the strong results achieved with the proposed approach. In the case of the top 2,000 ICD codes, we show that, although we are including almost more 600 codes, the performance is similar to that which was reported in the previous study in the area.

**Table 5:** Comparison between previous work and the “best” model from Table 2, considering two different scenarios (i.e., the ICD label set from previous work and the top 2,000 ICD codes in the new dataset). Results for the model marked with * were taken directly from the article by Coutinho and Martins [7].

	ICD level	Accuracy	Macro-averages		
			Precision	Recall	F1-score
Previous work *	Chapter	90.1	74.9	70.6	72.3
	Block	83.7	53.1	49.9	50.1
	Full-code	80.0	39.6	39.6	38.5
“Best” model (1,418 codes)	Chapter	92.8	78.6	81.8	79.9
	Block	86.2	60.3	60.5	58.7
	Full-code	81.8	47.9	47.1	45.3
“Best” model (2,000 codes)	Chapter	92.2	79.0	80.8	79.2
	Block	85.6	52.4	52.0	50.6
	Full-code	80.6	37.1	37.0	35.2

#### 4.3.4 Generating sets of ICD codes with coverage guarantees.

In order to assess the potential use of our model in a scenario where human coders can refine the automatically generated results [24], we explored the use of conformal prediction [9]. This is a simple approach for creating statistically rigorous uncertainty sets for the predictions of a model. Using this paradigm, we can assign individual instances to sets of ICD codes that are guaranteed to contain the ground-truth class with a user-specified probability. This means that, instead of predicting only one ICD-10 code per death certificate, we give as output the ICD-10 codes with higher probability, in order to achieve a desired level of coverage. The size of the resulting set of codes is determined by a score function and can be different for each instance, i.e., depending on the uncertainty of the model for each death certificate we can return a different number of codes as output. These computations do not involve additional training, instead just relying on a small amount of data, not seen during training, that is used as a calibration set. We followed the tutorial proposed by Angelopoulos et al. [9] and performed different experiments varying the parameter α∈[0,1], which is the user-chosen error rate corresponding to 1 − coverage. After an initial analysis, we set the parameter α=0.1.

**Fig 4:**
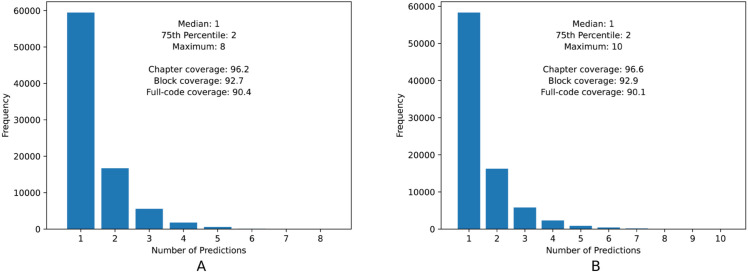
Frequency of instances per number of predictions in the conformal sets, when considering the conformal prediction strategy. (A) LLaMA-3.2-1B (fine-tuned). (B) LLaMA-3.2-1B (constrained decoding).

Fig 4 shows the frequency of instances for each possible size of the prediction set when considering α=0.1, for the fine-tuned model (which directly outputs a probability for each possible ICD code) versus the model that was only trained with causal language modeling. We additionally present the values of coverage per level of ICD specialization. The obtained results show that both types of models can effectively support a conformal prediction strategy, with similarly sized groups for the same coverage.

Regarding the chosen *α* value, we argue that despite results obtained with lower values of *α* could be more interesting, the predicted sets for each instance would be much larger, which is not the ideal situation. Therefore, we believe that α=0.1 is a good compromise between coverage, and keeping the size of the predicted sets relatively small. In both models, only one code is predicted per instance in the large majority of the cases, which would be interesting for a scenario in which human coders review a subset of the instances.

#### 4.3.5 Experiments with a dataset from a different time period.

To assess model generalization, we report results over an additional test set, referring to the last six months of 2021. We used our “best” model from Table 2 trained with data from 2014 to 2021 (excluding the last six months), and the results are given in Table 6. Although both the overall performance and the performance for the most frequent chapters slightly decreases, we can see that the model is still able to generalize well for more recent data.

**Table 6:** Results for the “best” model from Table 2 over the additional test set.

	ICD level	Accuracy	Macro-averages		
			Precision	Recall	F1-score
All Chapters	Chapter	89.4	75.0	68.8	70.2
	Block	80.2	32.2	32.7	30.7
	Full-code	74.7	16.8	17.6	16.0
Chapter II	Block	92.6	63.2	61.2	60.8
	Full-code	88.4	31.4	32.1	30.4
Chapter IX	Block	75.0	62.0	53.4	55.2
	Full-code	69.6	29.7	26.5	25.7

We also performed an additional test, considering the 64 ICD codes (and 71 associated instances) that were present in this additional dataset and not featured in the main set, and which were initially excluded. In this case, the accuracy at the level of full-codes drops to 74.6%, probably due to the fact that, although the model is able to generate the descriptive title of these codes, they are indeed rare and in most cases confused with other codes (i.e., only one of these new instances is correctly classified).

**Fig 5:**
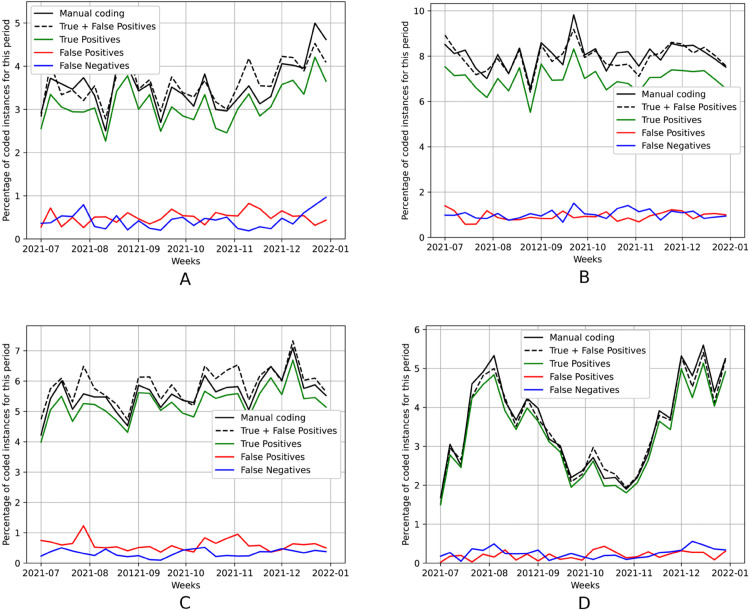
Percentage of weekly deaths in the last six months of 2021 for four specific causes of death obtained with the “best” model from Table 2. (A) Ischemic heart diseases (I20-I25). (B) Cerebrovascular diseases (I60-I69). (C) Influenza and pneumonia (J09-J18). (D) COVID-19 (U07).

Following previous work [6,7], we also tried to assess whether the model could support the monitoring of the prevalence of specific causes of death in near real-time, with a low error. Fig 5 presents time-series plots showing the weekly evolution of the percentage of deaths occurring in the last six months of 2021, associated to specific causes, namely ischemic heart diseases, cerebrovascular diseases, influenza/pneumonia, and COVID-19. The black solid lines correspond to the percentage of occurrences per week, as assigned manually. In order to represent the percentage of occurrences estimated by our automated method, we used Probabilistic Classify and Count (PCC) as our quantification method [10,11]. Instead of counting the number of certificates classified into one specific disease (i.e., classified into a set of specific ICD codes), we can sum the posterior probabilities returned by the classifier for these codes and for all the documents in the set, and then divide by the total number of documents. PCC usually outperforms the baseline Classify and Count (CC) method, which has already been verified experimentally by several studies [25]. We also verified that the error associated to the PCC method, when compared with the ground-truth, is slightly lower than the error for CC. Thus, the black dashed lines correspond to the percentage of occurrences estimated by PCC, and we also show the true positives (in green), the false positives (in red), and the false negatives (in blue).

The COVID-19 scenario (Fig 5D) is particularly interesting to analyze. Despite having a significant prevalence in this set, this code corresponds to a pandemic that only had its first cases in 2020, and therefore it has considerably less training data than other groups of diseases. Focusing on this case, we can see that the number of false negatives is almost negligible. The number of false positives is also low, although it slightly increases in the last months of 2021, due to the model assigning codes for other respiratory diseases. Still, the automated results provide a good approximation of the true values, which also occurs in the other groups of diseases.

#### 4.3.6 Application to other tasks.

We explored two distinct strategies to adapt generative language models to the ICD coding task, including one where we first adapt the model to the clinical domain by running a continual pre-training phase, followed by fine-tuning the model into a discriminative classifier, and another where we only use the language modeling objective, followed by constrained decoding at inference time. Experimental results show that the second alternative achieves similar classification results, at the same time presenting other advantages. On one hand, it requires considerably less training time. On the other hand, this model is also able to solve other related tasks through language generation, with one example being the prediction of ICD codes for auxiliary causes of death.

To exemplify the aforementioned claim, Table 7 presents the results for the “best” model in Table 2, in a multi-label classification task of predicting multiple causes of death, i.e., all the codes associated with a death certificate and not only the ICD code for the UCOD. These results were also obtained using constrained decoding at inference time. While we cannot establish a comparison with previous work regarding this specific task, the results confirm that we can use our model, without any architectural customization, in other related tasks. In future work, we plan to further explore this and also other tasks, such as the generation of explanations for the attribution of specific ICD codes.

**Table 7:** Results for the “best” model from Table 2 when considering the task of multi-label classification for multiple causes of death.

ICD level	Number of codes	Micro-averages			Macro-averages		
		Precision	Recall	F1-score	Precision	Recall	F1-score
Chapter	22	62.6	93.3	74.9	56.1	83.5	65.4
Block	1,648	52.6	86.4	65.4	24.3	30.0	25.4
Full-code	5,944	49.3	81.8	61.5	12.4	15.9	13.0

## 5 Conclusions and future work

We proposed different strategies to effectively use generative language models in the automatic assignment of ICD-10 codes to the UCOD from the textual contents of Portuguese death certificates, comparing the obtained results with those from a previously proposed BERT-type model [7]. Experimental results show that our approaches for training generative language models, so that they become capable of addressing this task, can achieve strong results, with a classification accuracy that is at least in line with the results obtained using encoder-only approaches, while offering other advantages.

The proposed approach can be integrated into a real-world ICD coding workflow, for instance as part of an human-in-the-loop pipeline. Mortality data can be first fed into the generative ICD coding model, which generates ICD code suggestions. These outputs can be used as approximate mortality statistics, or they can be subsequently reviewed through an interactive interface where human coders can approve or modify the proposed codes, considerably alleviating the burden to public health professionals. Both these strategies are currently being considered at the Portuguese DGS. In interviews and meetings with the clinical coders working for the DGS, we observed a good overall satisfaction with the use of software prototypes that integrate the ICD coding suggestions produced with our “best” model. The clinical coders have noted that the suggestions for the UCOD are easy to incorporate into their workflow, effectively supporting their activities and improving efficiency. They have also expressed a keen interest in being able to interact with the ICD coding model through questions in natural language.

The proposed approach also presents certain limitations. For instance, while it can be adapted to other languages or to more recent ICD versions, such as ICD-11, this likely requires retraining with appropriately labeled data.

For future work, one interesting idea is to train the language model with an even larger dataset, extended in order to include new related tasks. One possibility is to consider a specific additional task, with data produced with a rule that covers the challenge of distinguishing between the ICD codes most likely to correspond to the UCOD, given a set of possibilities. In fact, we can combine this idea with the conformal prediction approach and, with the same model, we can first predict the ICD codes with highest probability and then select the final code.

We can also pursue the idea of generating plausible explanations justifying the ICD code selected for the UCOD, e.g. prompting the model to explain its reasoning behind the coding decision. The model should ideally be able to explain to the user why it chooses a certain ICD code as the UCOD (e.g., over alternative auxiliary codes), according to the ICD guidelines. In fact, not only an explainable model has the potential to increase the users’ trust in automated approaches, but the explanations can also be important to identify missed or erroneous coding.

## Supporting information

S1 Appendix Language model prompts. (PDF)
