# Supplementary material for: ICD coding of death certificates with generative language models
Source: PLOS Digit Health. 2026 Feb 24;5(2):e0001245. doi: 10.1371/journal.pdig.0001245 (PMC12931742; doi:10.1371/journal.pdig.0001245)
Supplement: S1 Appendix — (PDF) [file pdig.0001245.s001.pdf]

S1 Appendix
Language model prompts

We created a set of rules to adapt the dataset of death certificates into a large set of textual instructions to train the language models. Each instance in the resulting dataset should contain a short context about the coding scenario, and all information about the deceased individual, along with a task instruction and an expected output. Since we opted by using the instruct versions of the LLaMA models for our experiments, it is essential to transform the prompt into a structured chat template to align with the initial training paradigm. Modern language models are typically trained to follow instructions within conversational formats, so structuring the input as a dialogue ensures the model interprets the prompt as an explicit request. Therefore, the final prompts start with a system description that gives a short context about ICD coding. Then, the user gives the information contained in the death certificate, together with a task instruction. Finally, the assistant returns the expected output.

The different rules that were created correspond to different tasks that we can envision, exploring the content of the death certificates. For instance, the main task concerns, given a death certificate and all the available additional information, predicting the ICD code for the UCOD. Alternatively, we can also ask the model to generate a death certificate given the ICD code for the UCOD. In addition to these examples, there are also several other related tasks, which we summarize in S1 Table. Using these different rules, models can be trained using many examples from the same death certificate, resulting in a large dataset with more than 2 million cases.

S1 Fig illustrates a prompt for one of the proposed rules, in which we ask the LLM to generate the ICD code for the UCOD. We present the text of an English translation for readability purposes, although we use Portuguese to train the model. Regarding the output, we expect the LLM to return not only the ICD code, but also its complete descriptive title. We argue that it is probably easier for the LLM to learn to generate a text description, rather than just a code, since in some cases the death certificate even contains words in the ICD descriptive title. For creating a full-title, we take advantage of the ICD hierarchy and we concatenate the title of the code itself, and the title of the chapter, sub-chapter, and parent codes (if those exist).

Regarding the LLaMA models, we used these rules for continual model pre-training. In the case of model fine-tuning, we also had to transform the original documents into a set of textual instructions. In fact, the input prompt is very

S1 Table. The proposed rules for generating the prompts for language model pre-training.

	User input	Assistant
Rule 1	Death certificate, clinical bulletin, autopsy report, and/or other annotations	ICD code for the UCOD
Rule 2	ICD code for the UCOD	Death certificate, clinical bulletin, autopsy report, and/or other annotations
Rule 3	Death certificate, clinical bulletin, autopsy report, and/or other annotations	ICD codes for the UCOD and also auxiliary causes of death
Rule 4	ICD codes for the UCOD and also auxiliary causes of death	Death certificate, clinical bulletin, autopsy report, and/or other annotations
Rule 5	Only clinical bulletin and/or autopsy report	ICD codes for the underlying and auxiliary causes of death
Rule 6	Death certificate (asking the model to specifically pay attention to one of the auxiliary causes)	ICD code for the specific auxiliary cause of death
Rule 7	Death certificate, clinical bulletin, autopsy report, and/or other annotations together with the ICD code for the UCOD	Explanation for the ICD code selected for the UCOD

similar to the one used for pre-training (S1 Fig), but we exclude the last paragraph with the user request part of the instruction, and also the assistant part. In this case, we also duplicate the information about the deceased individual (i.e., the user part), in order to emulate a bidirectional self-attention mechanism when building input representations. In regard to the experiments with the BERT model, we used the same prompts for continual model pre-training and fine-tuning, namely by using only the user part and excluding the request instruction. In this case, we did not have to duplicate the prompts, since the model already has a bidirectional context.

System

The assistant is an automatic system for analyzing death certificates. Its purpose is to ensure the quality and accuracy of information on mortality, assisting in the precise coding of causes of death into statistical categories, in accordance with the rules of the International Classification of Diseases (ICD) of the World Health Organization.

User

In the context of the automatic coding of death certificates, consider the following chain of events that led to the death of a gender individual with age years of age, with the immediate cause listed first and the underlying cause listed in the lowest line.
Main chain of events (Part I)
Also consider that other significant illnesses, conditions, or injuries have been reported as contributing to the death, but were not part of the primary causal sequence leading to the death.
Other conditions (Part II)
A clinical bulletin was also added, describing relevant information about the individual.
Clinical bulletin
Additional information is described in the autopsy report.
Autopsy report
Other relevant other annotations were also recorded.
Other annotations
Carefully reflecting on all the information, which International Classification of Diseases (ICD) code should be assigned as the underlying cause of death?

Assistant

Full-title - ICD code.

S1 Fig. Example of a prompt used for model training. The text that must be taken from the original documents is in red. The instruction for the LLM is shown in blue.
